# Genes and Environment: providing open access to environmental mutagenesis and genomics studies for global cooperation

**DOI:** 10.1186/s41021-015-0007-7

**Published:** 2015-06-16

**Authors:** Takashi Yagi

**Affiliations:** Department of Biology, Graduate School of Science, Osaka Prefecture University, 1-2 Gakuen–cho, Naka–ku, Sakai, Osaka 599-8570 Japan

**Keywords:** Environmental mutagen, Environmental genomics, Regulatory science, Pollution, Open access

## Introduction

*Genes and Environment* has been published since 2006, following the prior title *Environmental Mutagen Research*, the publication of which began in 1978 as the official journal of the Japanese Environmental Mutagen Society (JEMS). The aims and scope of the journal are to accelerate communication among scientists working globally in the field of genes and environment. The journal publishes articles across a broad range of topics including environmental mutagenesis and carcinogenesis, environmental genomics and epigenetics, molecular epidemiology, genetic toxicology and regulatory sciences. *Genes and Environment* was first published quarterly as a print journal, and all articles were freely accessible on the journal website. As a result of extensive discussion, JEMS concluded that a better platform to disseminate research and to share it with scientists globally is required due to great progress being made in the field of gene and environment research, particularly in Asia. To achieve this, JEMS decided that open access to all articles would be provided via the major platform, BioMed Central. 

Some Asian countries have experienced adverse effects of environmental pollutants on human beings and ecosystems for several decades, and now many Asian countries are facing various issues regarding environmental pollution, mainly due to rapid economic expansion. These past experiences should provide lessons for other countries. Scientists should be able to access research papers on environmental pollution and their health effects freely. Furthermore, some recent pollution is not just an issue for one country, but also for surrounding countries. We need a common platform for scientists globally to collaborate and to resolve these gene and environment issues. I introduce some of my personal experiences on these issues and comments in the following sections.

## Environmental pollution in everyday life

In the middle of February and March, people in Japan are filled with happy expectations for the coming blossom season, but many Japanese people, including me, suffer greatly from allergic rhinitis (pollinosis). This allergic rhinitis is caused by pollen of Japan–endemic cedar trees, *Cryptomeria japonica*. The increase in this allergy in the last few decades indicates that other factors, in addition to pollen, are involved as causative factors in this illness. Many studies have reported that the onset of allergic rhinitis is related to atmospheric pollution [1]. The atmospheric pollutant consists of suspended tiny particulates containing various aryl hydrocarbons that originate from vehicle and factory exhausts. The suspended particulate matter (SPM) can bind to cedar pollen and potentiate its allergic activity as an adjuvant. Pollen bound with SPM, especially that smaller than 10 nm (PM10) and 2.5 nm (PM2.5), is taken in by breathing, which leads to a runny nose and cough every spring in Japan. PM2.5 is a carcinogen classified as group 1, which indicates that sufficient evidence of its carcinogenicity in humans has been obtained by the International Agency for Research on Cancer (IARC) [2], and PM2.5 is supposed to contribute to an increase in lung cancer incidence [3].

**Fig. 1:**
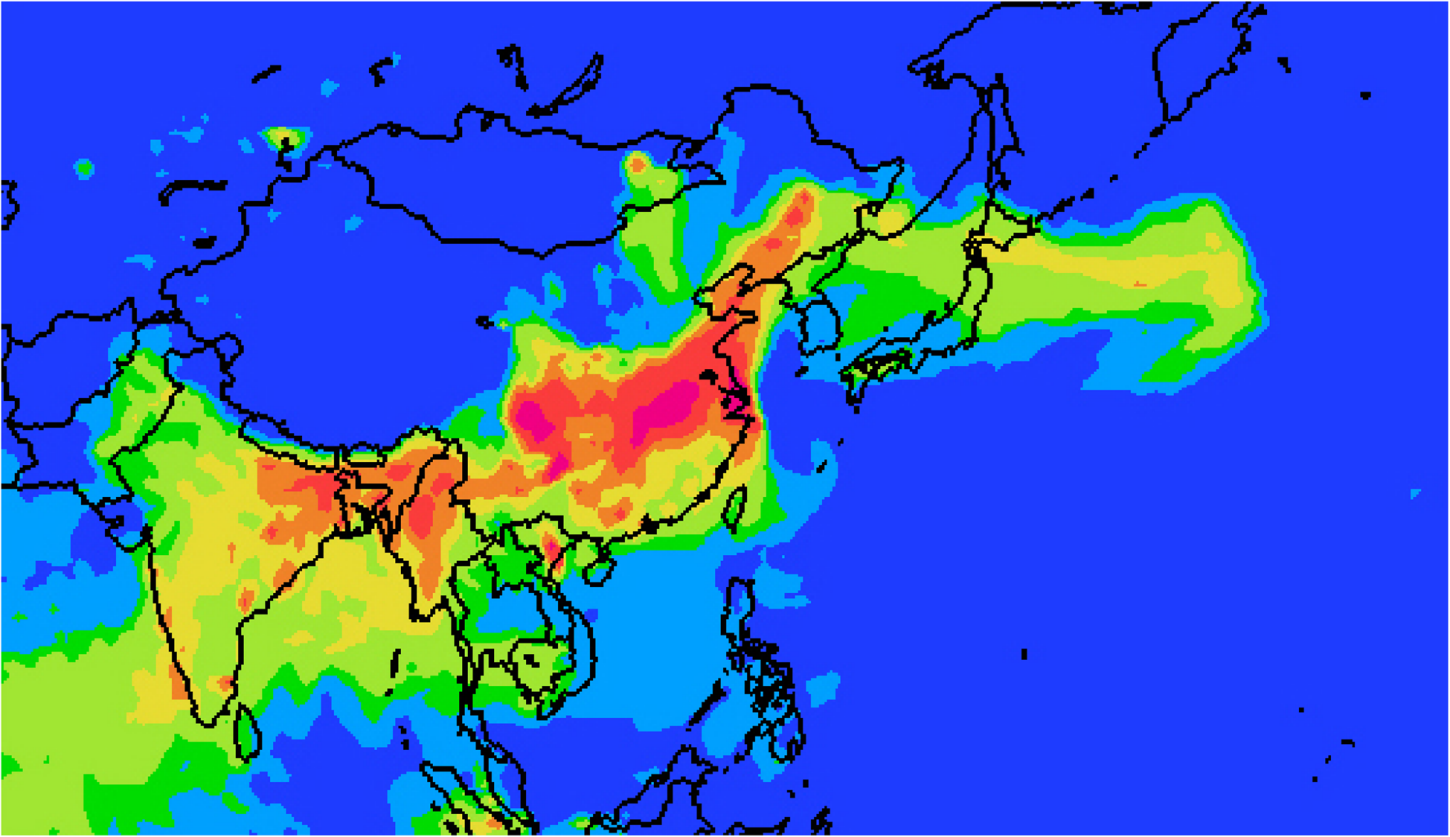
Concentration of PM2.5 in the atmosphere in East Asia on March 28, 2015. Data are obtained from a website on PM2.5 [8]. Areas of red, yellow, green, and light blue show increasingly high concentrations of PM2.5 in the atmosphere

When I attended the annual congress of the Environmental Mutagen Society of India in December 2013, my plane flew from Tokyo over the East China Sea in a clear blue sky, and then entered the airspace above the Asian mainland in Shanghai. After that, I found that the view of the Asian mainland from the plane was continuously hazy until I saw the sunset above the Indian Subcontinent. My expectations of seeing the beautiful Yungui Plateau and the Mouth of the Ganges were not fulfilled. This hazy weather spreading over a wide area was due to PM2.5 in the atmosphere, and this air pollution has been transgressing national borders eastward with the Asian monsoon (Fig. 1).

## Occurrence of occupational exposure

The environment has been cleaner since the beginning of the 21st century in Japan. This is due to the establishment of environmental standards for various chemicals, reflecting the heavy pollution and health problems in the previous century. Most sources of factory effluent and domestic wastewater have now disappeared after the removal of contaminants at treatment facilities. Junior high school students learn about the four major pollution–caused diseases in Japanese history: Minamata and the 2nd Minamata methyl–mercury poisoning in Kumamoto and Niigata in the 1950s–1960s, respectively; Itai–Itai disease due to chronic cadmium poisoning in Toyama in the 1960s; and Yokkaichi bronchial asthma due to factory smoke in Mie in the 1960s [4]. Such pollution–caused diseases are generally considered as past incidents, of which only memories are left.

Against this background, the news of pollution–induced cancers in my conurbation astonished me in 2005 and 2012. One was mesothelioma occurring in workers at a factory and its nearby residents [5]. The factory had produced building materials using asbestos until a national law banned their use in 2006. Although scientists had learned that asbestos is a carcinogen (IARC group 1), the decree from the government prohibiting its use came too late. The other was cholangiocarcinoma in workers at a printing company [6]. These workers had been washing away ink adhered to offset presses with 1,2–dichloropropane and dichloromethane for many years. After this incident, IARC raised the carcinogenicity rank of 1,2–dichloropropane from group 3 (not classifiable as to its carcinogenicity to humans) to group 1 (carcinogenic to humans), and that of dichloromethane from group 2B (possibly carcinogenic to humans) to group 2A (probably carcinogenic to humans) [2].

These incidents have taught us many lessons: i) Although legally binding regulations are enforced, there are still many loopholes in localities; ii) Even when an environmental standard has been set at the time of introduction of a chemical, cancer can still be induced later in people exposed to it; iii) There are still many environmental chemicals of which the carcinogenicity is not clear.

## Genes and Environment for the exploitation of local experiences

In many Asian countries, economic development seems to take precedence over environmental preservation, and residents are exposed to environmental toxicants and suffer adverse effects on their health. Other countries underwent similar experiences decades ago, and lots of scientific research has consequently been carried out. However, not all research papers describing them have been made available to the public; only those who belong to institutes or universities that pay subscription charges for accessing journals can read these papers.

A country confronting adverse environmental influences should learn from the previous experiences of other countries. There is a Japanese proverb that “one man’s fault is another’s lesson”. To resolve these environmental issues, we need a platform to share information and to access and use anyone’s data freely based on a creative commons license [7]. Such a platform provides opportunities for international discussions and cooperation to resolve these issues. Elucidation of the mechanisms causing environmental pollution and affecting human health is also important on such a platform. *Genes and Environment* is the first fully open access journal dedicated to environmental mutagenesis and genomics, and the journal also covers research from broad fields related to genes and environment. All data in the journal articles can be accessed and utilized freely by anyone under the condition of a creative commons license.

I hope that the journal will become a new platform for collaborative studies on genes and environment, and greatly contribute to the health and welfare of people globally. I am looking forward to disseminating many important papers from all over the world.
